# Composite Hydrogels Based on Bacterial Cellulose and Poly-1-vinyl-1,2,4-triazole/Phosphoric Acid: Supramolecular Structure as Studied by Small Angle Scattering

**DOI:** 10.3390/biomimetics8070520

**Published:** 2023-11-02

**Authors:** Ruslan Y. Smyslov, Artem I. Emel’yanov, Ksenia V. Ezdakova, Svetlana A. Korzhova, Yulia E. Gorshkova, Albert K. Khripunov, Alexandra V. Migunova, Natalia V. Tsvigun, Galina F. Prozorova, Varvara O. Veselova, Gennady P. Kopitsa, Lijun Lu, Yanchao Mao, Alexander S. Pozdnyakov

**Affiliations:** 1 Institute of Macromolecular Compounds RAS, NRC KI, 199004 Saint Petersburg, Russia; urs@macro.ru (R.Y.S.); biocell@macro.ru (A.K.K.); 2 Petersburg Nuclear Physics Institute NRC KI, 188300 Gatchina, Russia; matissa.vkv@gmail.com (K.V.E.); kopitsa_gp@pnpi.nrcki.ru (G.P.K.); 3 A.E. Favorsky Irkutsk Institute of Chemistry, Siberian Branch, Russian Academy of Sciences, 664033 Irkutsk, Russia; emelyanov@irioch.irk.ru (A.I.E.); korzhova@irioch.irk.ru (S.A.K.); prozorova@irioch.irk.ru (G.F.P.); 4 Joint Institute for Nuclear Research, 141980 Dubna, Russia; yulia.gorshkova@jinr.ru; 5 Institute of Physics, Kazan Federal University, 420008 Kazan, Russia; 6 Department of Microbiology, Biological Faculty, Saint Petersburg State University, 199178 Saint-Petersburg, Russia; sasha_mig_2405@mail.ru; 7 Federal Scientific Research Centre “Crystallography and Photonics” of the Russian Academy of Sciences, 111933 Moscow, Russia; n_tsvigun@mail.ru; 8 Kurnakov Institute of General and Inorganic Chemistry RAS, 119991 Moscow, Russia; ibvarvara@yandex.ru; 9 I.V. Grebenshchikov Institute of Silicate Chemistry RAS, NRC KI, 199034 Saint Petersburg, Russia; 10 Key Laboratory of Materials Physics of Ministry of Education, School of Physics and Microelectronics, Zhengzhou University, Zhengzhou 450001, Chinaymao@zzu.edu.cn (Y.M.)

**Keywords:** interpenetrating polymeric networks, cellulose composite, SANS, USANS

## Abstract

New composite hydrogels (CH) based on bacterial cellulose (BC) and poly-1-vinyl-1,2,4-triazole (PVT) doped with orthophosphoric acid (oPA), presenting interpenetrating polymeric networks (IPN), have been synthesized. The mesoscopic study of the supramolecular structure (SMS) of both native cellulose, produced by the strain *Komagataeibacter rhaeticus*, and the CH based on BC and containing PVT/oPA complex were carried out in a wide range of momentum transfer using ultra- and classical small-angle neutron scattering techniques. The two SMS hierarchical levels were revealed from 1.6 nm to 2.5 μm for the objects under investigation. In addition, it was shown that the native BC had a correlation peak on the small-angle scattering curves at 0.00124 Å^−1^, with the correlation length ξ being equal to ca. 510 nm. This motive was also retained in the IPN. The data obtained allowed the estimation of the fractal dimensions and ranges of self-similarity and gave new information about the BC mesostructure and its CH. Furthermore, we revealed them to be in coincidence with Brown’s BC model, which was earlier supported by Fink’s results.

## 1. Introduction

One task facing materials science is the search for new matrices to create composites with unique properties required for applications in medicine (such as radiological treatments and photodynamic therapy of cancer), optoelectronics, construction materials and protective clothing. Bacterial cellulose (BC) is an exopolysaccharide produced by gram-negative bacteria from genera, such as *Komagataeibacter* (formerly *Acetobacter*) and *Agrobacterium*, as well as *Enterobacter*, *Achromobacter*, *Azotobacter*, *Rhizobium* and *Salmonella* [1]. It is important to note that BC is produced by gram-negative bacteria, such as *Sarcina ventriculi* [1]. The BC nanogel film (NGF) forms a pellicle on the surface of the nutrient medium under motionless conditions. This pellicle is capable of retaining water at a ratio of approximately 1:100 (dry matter to water). NGF is widely used as a matrix for the preparation of various organic-inorganic composites after pre-treatment due to its specific physicochemical properties and unique structure. For example, BC can serve as a rigid scaffold for the formation of interpenetrating polymer network (IPN) composite hydrogels (CH) to produce artificial cartilage [2].

Recently, interest in organic-inorganic composites has grown tremendously. It relates to potential applications of these hybrid materials in different areas like optoelectronics, nanomedicine, cosmetics, textile industry, etc. [3,4,5]. Cellulose-based composites have intrinsic biodegradability and biocompatibility, which makes them more attractive in perspective. In particular, the cellulose produced by *Komagataeibacter rhaeticus* is remarkably different compared to the structure of the cellulose obtained from wood, cotton or a species of algae—*Valonia ventricosa* [6,7,8].

Previously [9,10], we investigated the size and phase composition of Se^0^ or Ag^0^ nanoparticles stabilized by polymer in nanogel films of bacterial cellulose using SAXS, electron diffraction and electron microscopy. We used the notions of R. Malcolm Brown Jr. [11] and Fink H-P [12] and proposed the network model of BC intercalated by drug nanoparticles. Therefore, BC can function as a scaffold material for the regeneration of a wide variety of tissues that may eventually become an excellent platform for medical nanotechnologies [3,13].

As we have shown previously [2], BC can be used as an excellent matrix for the mechanical reinforcement of hydrogels based on polymers, such as polyacrylamide. The presence of hydrophilic synthetic polymers in a material gives it elasticity and creates the required degree of swelling, while the rigid-chain framework, namely cellulose, gives strength to the composite. The composite hydrogels reinforced by BC are close to various types of natural articular cartilage in water content and the level of mechanical characteristics and for these reasons can be used for damaged cartilage replacement. In addition, in our work [14], ultra-small-angle neutron scattering (USANS), small-angle neutron and X-ray scattering (SANS and SAXS), as well as low-temperature nitrogen adsorption, were used in the studies of micro- and mesostructure of the polymer matrix prepared from air-dry preliminarily disintegrated cellulose NGF and the composites based on the non-native but disintegrated BC. The composites included ZrO_2_ nanoparticles, Tb^3+^ in the form of low molecular weight salt and a metal–polymer complex with poly(vinylpyrrolydone)-poly(methacryloyl-*o*-aminobenzoic acid) copolymer. The combined analysis of the data obtained revealed three levels of fractal organization in BC mesostructure and its composites. It has been shown that both the composition and the aggregation state of dopants have a significant impact on the structural characteristics of the organic-inorganic composites. The above proposed model of the organization of the micro- and mesostructure of hybrid organic-inorganic composites based on the polymer matrix of air-dry pre-disintegrated BC nanogel film has been confirmed by the analysis of the experimental data obtained by low-temperature nitrogen adsorption. The obtained values of specific surface area, *S*_BET_, in the range of 69.6–330 m^2^/g clearly indicate that these composites have a porous structure with a sufficiently developed system of open pores [14].

The addition of lanthanides into the hybrid polymer matrix can create materials with tunable or modulated properties [5,15]. Lanthanide ions have unique photophysical properties, especially with respect to their applications in photonics [16,17,18], generation and amplification of light in lasers [19], or optical amplifiers—e.g., Er^3+^-doped fiber amplifiers, EDFA’s [20,21]. Promising applications, such as light-emitting devices, active waveguides in the visible and near-IR spectral regions, active coatings, and bio-medical actuators and sensors, may also be envisaged. They open up exciting directions in materials science and related technologies with significant implications for the integration, miniaturization and multi-functionalization of devices. Thus, BC membranes with indium tin oxide and SiO_2_ can be used as flexible substrates for the fabrication of organic light-emitting diodes [22]. In our work [14], the composites based on BC and containing Tb^3+^ ions demonstrate efficient luminescence; its intensity is an order of magnitude higher in the case of the composites with the metal–polymer complex. It has been found that there is the optimal content of ZrO_2_ nanoparticles in composites, resulting in increased Tb^3+^ luminescence.

The fundamental problem at the present stage of the development of concepts about cellulose from different evolutionary sources is to understand how its supramolecular structure (SMS) is formed under native conditions. Indeed, regenerated cellulose no longer forms the same SMS as observed in living nature. Using small-angle scattering (SAS) at the mesoscopic size level, attempts have already been made to understand SMS for native BC. Thus, in [23], the authors considered cellulose ribbons comprising a core containing a water-impermeable crystalline backbone, which is surrounded by a network of paracrystalline cellulose together with bound water, and a shell containing only paracrystalline cellulose and water.

In addition, Koizumi (2008) investigated the domain hierarchy in native bacterial cellulose with respect to amorphousness over a wide range of length scales, from nm to 10 μm using small-angle neutron scattering (SANS). Koizumi et al. [24] interpreted SANS from three different hierarchical structures in bacterial cellulose, namely a polymer gel network, a single bundle composed of cellulose nanoribbons, and concentration fluctuations of the centers of mass of the crystallites composing bundles. According to the data of SANS quantitative analysis based on the ideas of mass fractal and its physical size constraints, it was found that 90% of cellulose bundles consist of cellulose.

With the aid of spin-echo small angle neutron scattering, we have previously investigated the interpenetrating polymeric network of the composite hydrogel composed of BC and polyacrylamide [25]. An analysis of the sample in a fixed orientation revealed density inhomogeneities, with a characteristic scale of 11.5 ± 0.5 μm when spin-echo length was aligned with the growth plane of BC. The detected uniaxial anisotropy has been found to be associated with tunnel-like formations in BC. In addition, we proffered the results about synthesis [26] for the state-of-the-art composite material composed of BC and poly-1-vinyl-1,2,4-triazole (PVT) with Cu^2+^. The stabilization of the hybrid system is ensured by the formation of coordination bonds between Cu^2+^ and PVT nitrogen atoms in the fourth position of a monomeric unit. PVT is one of the promising polymers for medical use. It is highly soluble in water, has a controlled molecular weight (10^4^–10^6^ kg/kmol), is resistant to acid and alkaline hydrolysis, and is nontoxic (LD_50_ > 5000 mg/kg). According to the results of comparative morphological studies of animal organs and brain tissues, intragastric administration of PVT does not cause any structural changes in the body [27]. PVT is characterized by high complexing ability. Due to the presence of nitrogen atoms with a lone electron pair in the fourth position of the hetero ring, it easily forms complexes with atoms of various metals and has a high stabilizing ability when forming polymer nanocomposites.

The monomer 1-vinyl-1,2,4-triazole (VT) ensures the formation of narrowly dispersed polymers under RAFT polymerization conditions [28]. Under conditions of free radical polymerization, it forms functional copolymers with various comonomers [29,30]. Based on PVT, proton-conducting polymeric membranes with high ionic conductivity (up to 10^−2^ S/cm) at elevated temperatures (>100 °C) have been developed [31]. In work [26], the composite materials’ mesostructure was studied from 1.6 nm to 2.5 μm by small-angle and ultra-small-angle neutron scattering methods. It has been found that IPN complexes have three types of inhomogeneities: BC, PVT and PVT complex with Cu^2+^. In addition, three hierarchy levels of BC remain in the SMS of that hybrid material.

This article serves as a follow-up of our previous work [26] and presents the results of the synthesis and study of CHs, which are IPNs based on BC, as a rigid-chain polymer that imparts strength to the composition, and PVT complexed with orthophosphoric acid (oPA) as the second network component instead of the PVT complex with Cu^2+^. It is important to say that PVT plays a role as a flexible-chain hydrophilic polymer capable of providing samples with elasticity and coordination bonds with metal ions, as mentioned above. Our work aimed to investigate the SMS of native BC and first synthesized IPN based on it when using PVT complexed with oPA in a wide range of momentum transfers using small-angle neutron scattering. SANS was used to determine the supramolecular structure, which belongs to a higher level of hierarchy than the crystal structure of BC microfibrils. Considering the significant difference in the scattering length densities of BC crystallites and an amorphous polymeric part, to elucidate the hierarchical structure of the IPN, the objective was to find the contrast in the scattering length densities using D_2_O. This allowed us to reveal features in the hydrogel composites’ SMS by small-angle neutron scattering methods. The novelty of the obtained data included the fact that it was managed to find the near-order between BC nanoribbons at the mesoscopic level of study. In addition, the objectives of the study can be attributed to the intention to see what changes in the SMS of native BC occur during the interaction of the IPN components at the mesoscopic level of study.

## 2. Materials

According to the previously described method, 1-vinyl-1,2,4-triazole was synthesized as a starting reagent for the synthesis of composite hydrogels [27]. Azobisisobutyronitrile (AIBN, 99%, from Sigma-Aldrich, St. Louis, MO, USA), N,N’-methylene-bisacrylamide (MBA, from Fluka, Buchs, Switzerland), and orthophosphoric acid (OFA, 99%, from Merck, Darmstadt, Germany) were used without further purification. H_2_O was distilled. Argon with a purity of 99.9 was used in the reaction.

### 2.1. Synthesis of Bacterial Cellulose

BC was cultivated using *Komagataeibacter rhaeticus* strain CALU-1629 (EAPO Patent no. 201700517, 2017) from St. Petersburg’s State University (the Department of Microbiology) by an original technology (Patent RU №2141530, 1999; Patent RU №2189394, 2002). The BC was subsequently washed in water solutions of potassium hydroxide at 100 °C and then washed in water at room temperature to chemical purity. The resulting BC samples were gel-like pellicles with a thickness of up to 40 mm, containing ca. 99 wt.% of water.

### 2.2. Synthesis of a Composite Hydrogel

To synthesize CH, which is an interpenetrating polymer network, we prepared a composition consisting of VT monomer (6.51 g), AIBN, a radical type of initiator (0.5 wt%), and MBA, a low molecular weight crosslinking agent (0.2 mol %), which was stirred on a magnetic stirrer at room temperature until all components were completely dissolved. Then, a BC snippet of 1.5 × 1.5 × 1.0 cm^3^ was placed in this composition and kept for 8 h. The indicated BC sample saturated with the components of the composition (VT, AIBN, MBA) was fixed between two glass plates, placed in a glass bottle, and purged with argon. This bottle with sample BC was placed in an oven and held at 65 °C for 6 h. Under these conditions, VT polymerized and CH formed on the basis of BC and VT in the form of an interpenetrating network. The CH sample obtained was repeatedly washed with distilled water to remove the residual monomer and other low molecular weight impurities, placed in water, and swollen to an equilibrium state. The water content in CH was determined from the difference between the weights of the initial sample and the sample dried to a constant weight in a vacuum oven at 160 °C. The nitrogen content in CH was 40.51%. The CH samples swollen in water were doped with orthophosphoric acid by keeping these samples in homogeneous solutions of orthophosphoric acid at concentrations of 10% and 50% for 30 h.

## 3. Methods

For polymerization, a “BINDER” BD 53 thermal cabinet (Germany) was used. The composition and structure of the synthesized CH were studied using elemental analysis (C, H, N) performed on a Thermo Finnigan Flash EA 1112 automatic analyzer (Thermo Fisher Scientific, Cambridge, UK) and IR spectra, which were recorded on a Bruker Vertex 70 spectrometer (Bruker Corporation, Karlsruhe, Germany) in the range of 400–4000 cm^−1^.

### 3.1. SEM

The microstructures of the systems under investigation were studied using scanning electron microscopy (SEM). SEM images were obtained using a Tescan Amber GMH microscope (Tescan, Brno, Czechia). Images were collected at an accelerating voltage of 2 kV using an Everhart–Thornley secondary electron detector at a working distance of 6 mm. For SEM measurements, the samples were not specially treated (e.g., coated with conducting material).

### 3.2. XRD

X-ray diffraction (XRD) patterns were obtained on a powder diffractometer (D8 Advance, Bruker Corporation, Cu radiation) for air-dried BC, as well as BC/PVT composites obtained from CH based on PVT and BC.

### 3.3. Neutron Scattering

Small-angle neutron scattering (SANS) investigations were performed using a YuMO time-of-flight spectrometer at the IBR-2 pulsed reactor (Dubna, Moscow region, Russia). The data were collected in a two-detector configuration [32] in a *q*-range (momentum transfer) of 0.007–0.4 Å^−1^. The raw data treatment was performed using the SAS program [33]. The final small-angle neutron scattering curves are presented on an absolute scale with background subtraction [34]. The studied samples were placed in 1 mm thick quartz cells (Hellma, Germany). During data collection, the samples were kept in a temperature-controlled holder (± 0.2 °C) connected to the liquid thermostat (Lauda, Germany). The standard data acquisition time per sample was 30 min.

The very small-angle scattering techniques (VSANS) were carried out on a KWS-3 (FRM-II reactor, Garching, Germany). The KWS-3 is a high-resolution SANS diffractometer running on the focusing mirror principle [35,36]. The measurements were performed at neutron wavelength λ = 12 Å with Δλ/λ = 0.2. The range of momentum transfer 1.6 × 10^−4^ < *q* < 3.5 × 10^−2^ Å^−1^ was determined using two sample-to-detector distances (1 and 10 m). The scattered neutrons were detected by a two-dimensional position-sensitive detector (active area ∅ = 8.7 cm with spatial resolution 0.36 mm × 0.39 mm) based on ^6^Li glass scintillators. 

The raw data were corrected using standard procedures [37] considering the scattering from the setup equipment and packing materials. The resulting 2D isotropic spectra were averaged azimuthally, and obtained 2D spectra for two sample-to-detector distances were merged. Their absolute values were determined by normalization to the incoherent scattering cross section of the plexiglass with inclusion of the detector efficiency [37] and thickness *L*_s_ for each sample. All measurements were performed at room temperature. QtiKWS software (forked from QtiPlot v. 0.8.9, 2007) [38,39] was used for data processing.

With both types of facilities, we investigated neutron scattering for selected systems over a wide range of momentum transfer 1.6 × 10^−4^ < *q* < 0.4 × 10^−1^ Å^−1^. This corresponds to the sizes of the objects under study, from the maximum 3.9 μm to the minimum 1.6 nm. The overlap of the momentum transfer ranges of 0.7 × 10^−2^ < *q* < 3.5 × 10^−2^ Å^−1^ was sufficient (half a decade) to reliably combine the obtained small-angle scattering curves.

## 4. Results and Discussion

**Scheme 1:**
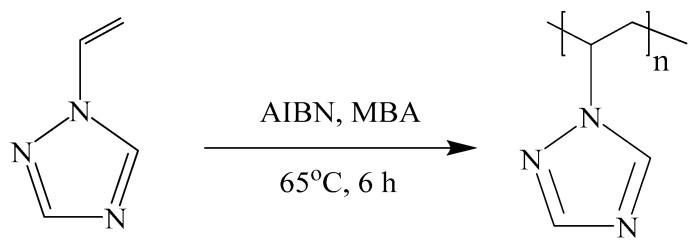
Polymerization of 1-vinyl-1,2,4-triazole inside the BC matrix.

In order to bring about the polymerization of 1-vinyl-1,2,4-triazole with the purpose of forming an interpenetrating network of PVT and bacterial cellulose, Scheme 1 was followed diligently.

The data on the composition of the synthesized composite hydrogels and their water content are given in Table 1.

**Figure 1:**
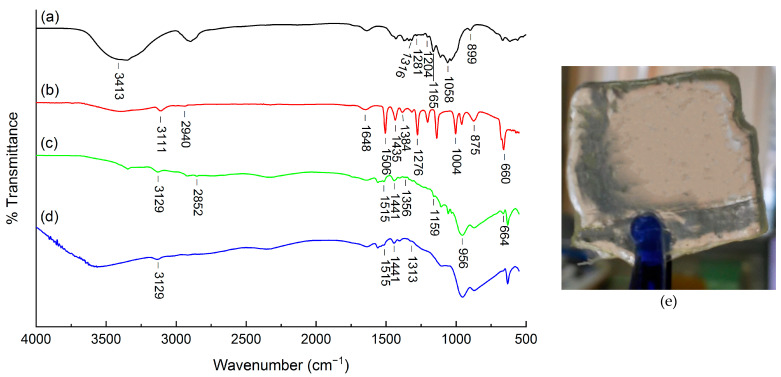
IR spectra of the BC (**a**), composite hydrogel (**b**), hydrogel doped with 10% oPA (**c**), hydrogel doped with 50% oPA (**d**). Picture of BC/PVT/oPA hydrogel (**e**).

The composition and chemical structure of the resulting composite materials were determined from the elemental analysis and IR spectroscopy data (Figure 1).

In the IR spectra of the obtained samples (Figure 1a,b) of composite hydrogels, there are absorption bands characteristic of both cellulose and poly-1-vinyl-1,2,4-triazole. Namely, the maxima inherent in cellulose were preserved (cm^−1^): 3413 (ν OH groups involved in intermolecular and intramolecular H-bonds), 2940 (νCH and CH_2_), 1648 (δ bonds HOH due to the presence of strongly bound water), 1435 (δ CH_2_), 1384 (δ OH in CH_2_OH), 1281 (δ CH_2_ in CH_2_OH), 1204 (δ OH), and 1165 and 1058 for ν C-O in CH_2_OH. The 899 cm^−1^ band confirmed the presence of β-1,4 bonds. The IR spectra also contained absorption bands corresponding to the stretching and bending vibrations of the triazole ring (cm^−1^): 3111 (С-Н), 1506 (C=N), 1435 (С-N), 1276 (N-N), 1004 (С-Н), and 660 (С-N).

**Scheme 2:**
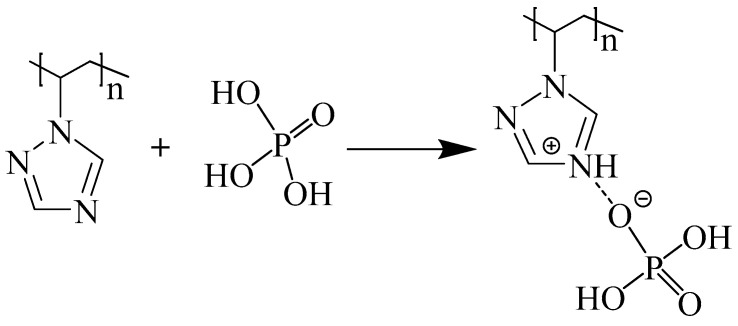
Interaction of PVT with phosphoric acid.

In the process of doping the obtained composite hydrogels with the use of phosphoric acid, the corresponding complexes of PVT and acid were formed (Scheme 2).

When analyzing the IR spectra of the initial composite hydrogels and hydrogels obtained after their doping with phosphoric acid, it was found that there was a significant shift and a change in the intensity of the absorption bands corresponding to vibrations of the triazole ring (Figure 1c,d). Namely, there was a shift of δ C–N from 660 to 664 cm^−1^, ν C–N (1435 cm^−1^) shifted to 1441 cm^–1^, and the absorption band ν C=N (1506 cm^–1^) shifted to the high-frequency region spectrum up to 1515 cm^−1^. The intensity of these bands changed greatly. In addition, bands appeared at 2852–3129 cm^−1^, which were related to the stretching vibrations of the N–H bonds of the protonated PVT fragments. Bands in the range 1356–1313 cm^−1^ referred to the P=O bonds, and the appearance of bands in the region 1159–956 cm^−1^ was characteristic of a mixture of HPO_4_^2−^ and H_2_PO_4_^−^ [40,41]. The presence of HPO_4_^2−^ and H_2_PO_4_^−^ anions means that phosphoric acid in the system is ionized, and the existing form of phosphoric acid is mainly not in the molecular state but in the ionic state [42].

Changes in the position of the absorption bands and their intensity confirm the occurrence of the proton exchange reaction between PVT in the composite hydrogel and phosphoric acid and indicate the protonation of the nitrogen atom in the 4th position of the heteroring (Scheme 2). The observed changes in the position of the absorption bands and their intensity confirmed the occurrence of the proton exchange reaction between PVT in the composite hydrogel and phosphoric acid and indicate the protonation of the nitrogen atom in the 4th position of the PVT heteroring (Scheme 2).

### 4.1. SANS and VSANS Studies of Native BC

**Figure 2:**
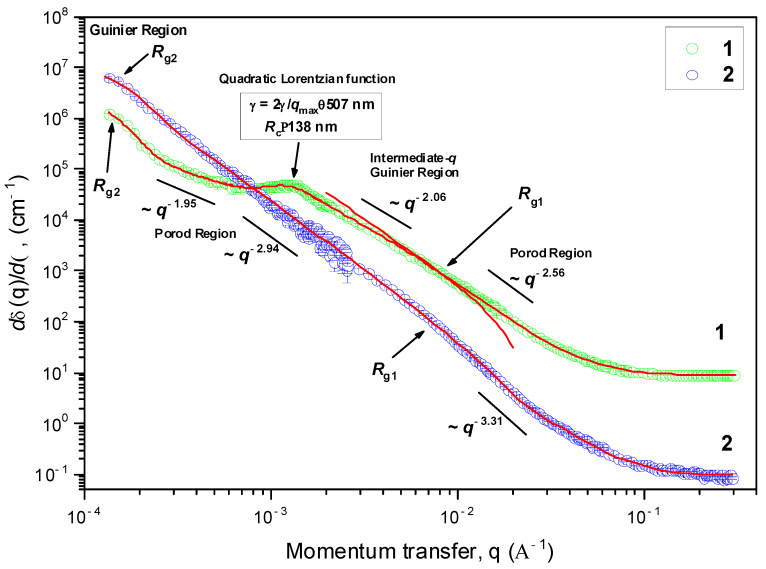
SANS and VSANS for BC nano-gel film (1) and dried BC (2). The red lines are the result of fitting the SAS data.

It is well known that BC microfibrils, which make up bacterial cellulose nanoribbons, have a crystalline structure [43]. For air-dried BC, we observed a characteristic diffraction pattern (Figure S4a in Supplementary Materials) corresponding mainly to cellulose Iα with a one-chain triclinic P1 unit cell [44]. Small-angle neutron scattering was used to determine the SMS, which belongs to a higher hierarchical level than the crystalline structure of the microfibrils. The small-angle neutron scattering method allowed us to study the hierarchical structure of native BC nano-gel film without distorting the supramolecular structure of the latter. The study included a method of contrast variation by replacing H_2_O with D_2_O, which allowed us to study the shape and inhomogeneities of supramolecular formations at different levels of hierarchy. The *q*-dependences of the SANS/VSANS cross-sections for the NGF of native BC are presented in Figure 2 in the log-log scale. 

It is well known [5,9,10,11,12], that BC contains 99% water and thereby synthesized samples also consisted of much water. Thus, the analysis of neutron scattering cross-section *dΣ(q)/dΩ* is impossible for large *q* ≥ 7 × 10^−2^ Å^−1^. This is caused by incoherent scattering on hydrogen atoms contained in these samples. Thereby, analyses of size and local structure of scattering heterogeneities are unfeasible.

As can be seen from Figure 2, the neutron scattering curves of the native cellulose show two sections obeying the power law and a well-defined peak. In the SANS experiment, the power-law dependence of the cross section is observed in a certain *q*-region *q* > 1/R, where *R* is the characteristic scale of a structure level of the system. At this time, its fractal dimension is determined by the exponent of power *n*, or, more precisely, by the deviation from the Porod asymptotic dependence (*n* = 4) [45]. Meanwhile, the crossover point *q_c_* allows calculation of the characteristic scale *R* of scattering heterogeneity of the observed structure level. This could be done by using the Guinier approximation [46,47].

Thus, the VSANS and SANS data for BC nano-gel film demonstrated a behavior that is typical for scattering of systems with hierarchical multilevel structure (Figure 2) [47,48]. The resulting data are presented in Table 2. Using the quadratic Lorentzian [49], we showed that for the native NGP of bacterial cellulose, there was a structural peak in the neutron scattering curve at *q_max_* ≃ 0.00124 Å^−1^ (see Equation (S4) in the Supplementary Materials). The correlation length ξ, which characterizes the near order, was equal to 2*π/q_max_* ≃ 507 (nm).

**Figure 3:**
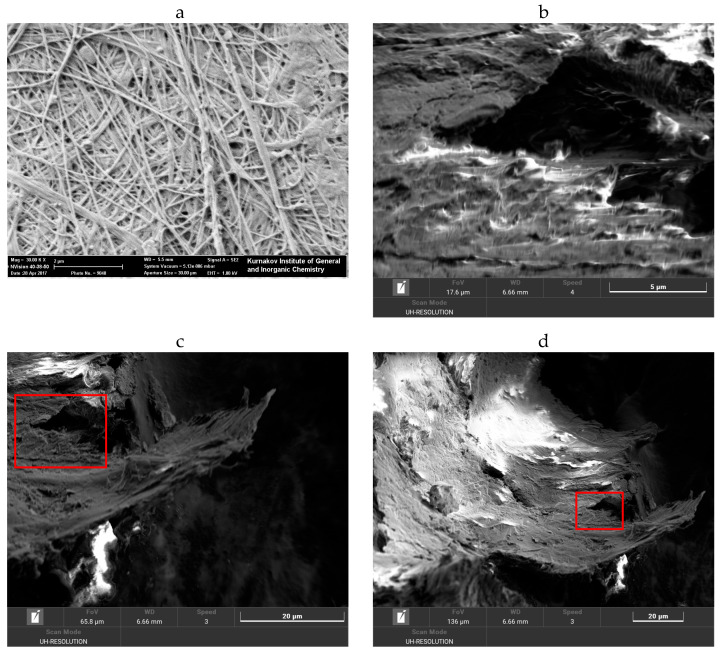
The SEM microphotograph for an air-dried nano-gel film of BC (**a**) and a composite of BC and PVT (**b**–**d**). In (**c**,**d**), the red rectangles depict the patch captured in Figure (**b**) at high magnification.

It should be stated that upon air drying of the BC nano-gel film, the structural peak disappeared (curve 2, Figure 2). In this case, the unified global scattering function (see Equation (S2) in Supplementary Materials) with two hierarchical levels was used to fit the SANS curve. As a result, the radius of gyration at the first hierarchical level was *R*_g1_ = (75 ± 3) nm, and at the second level, it was *R*_g2_ = (1.57 ± 0.1) nm. Estimation of the fractal aggregate size, $R_{c2}$, for the second level was as follows:

Rc2=Dm+2Dm1/2·Rg2,

where mass-fractal dimension: *D_m_* = *m*_2_ = 2.94 (Figure 2, curve 2). Thus, the size of such a fractal is {(2.94 + 2)/2.94}^0.5^·1.57 = 2.04 μm. It was formed by surface fractal clusters of the first level with dimensionality *D_s_* = 6 − *m*_1_ = 6 − 3.31 = 2.69, for which we obtained the characteristic size as $R_{c1}$ = {(2.69 + 2)/2.69}^0.5^·75 = 99 nm. In the SEM micrographs (Figure 3a) for air-dried BC, we matched the thickness of the nanoribbons with the value of $R_{c1}$, which formed a mass fractal with a maximum size of $R_{c2}$ = 2.04 μm.

**Figure 4:**
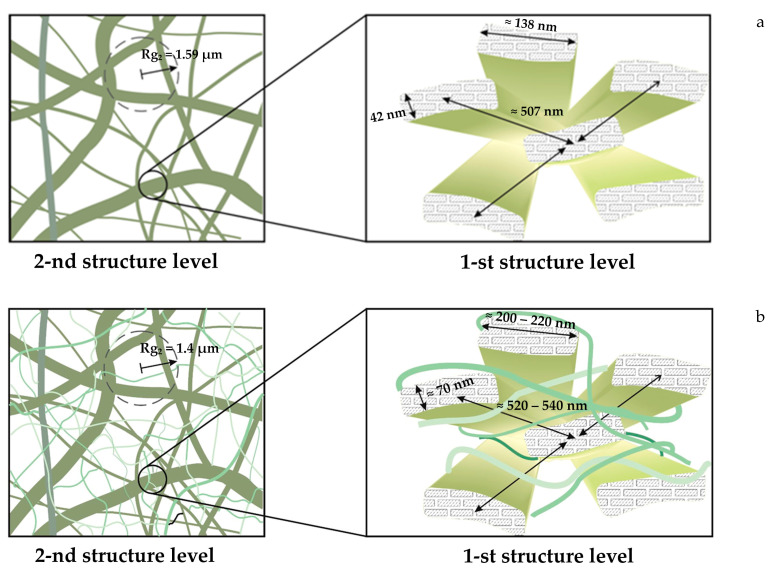
Schematic of the structure of native BC (**a**), interpenetrating BC/PVT network (**b**).

Based on the data shown in Table 2, we presented a plausible model of the NGP of bacterial cellulose in the native state (Figure 4a). In our experiment, we first detected the characteristic size and the width of the peak, with a characteristic size of *R*_c_ = 138 nm. It can be hypothesized that this size characterizes the width of the physical meshing of two nanoribbons—the overlap of them over each other in width. The width of one nanoribbon according to literature data up to 150 nm [24], so their overlap in width may well be of the order of 140 nm, considering the error of methods. The scattering curve in the interval of momentum transfer of 0.002–0.01 Å^−1^ is well approximated by the generalized Guinier/Porod law (see Equation (S1) in Supplementary Materials): the variable dimension, *s*, is ca. 2 (Table 2), indicating the lamellar structure of the scattering inhomogeneity.

The presence of this peak indicates a dense contact of BC lamellae due to the possible formation of multiple hydrogen bonds at the contact site. The distance between these physical links is about 500 nm, as indicated by the correlation length ξ = 507 ± 4 nm. Furthermore, going into the region of larger momentum transfer, if we assume that the scattering originates from the BC lamella cross-section, we see the thickness of either a single nanoribbon or their associate. This thickness is characterized by a radius of gyration, *R*_g1_, equal to 12.1 nm. If we assume that the minimum thickness of the ribbon is 6 nm, which is consistent with Brown’s model and does not contradict the ideas presented in works [23,24], then we can think that the observed radius of gyration *R*_g1_ of 12.1 nm really characterizes the thickness of the formation of two overlapping nanoribbons. Thus, we look at the layered structure from the side, which is formed by overlapping ribbons.

If going even further into larger momentum transfer, one can indeed see Porod’s scattering on wide nanoribbons of cellulose, which means scattering on large smooth surfaces of crystalline structures compared to the length of the incident radiation. Indeed, the width of the nanoribbon according to literature data can be up to 150 nm [23,24]: it can be seen in electron micrographs as well (Figure 3).

What happens if one goes to the region of a small momentum transfer from the observed peak at 0.00124 Å^−1^. It is said above that nanoribbons due to physical meshing, create cells with a characteristic size between nodes equal to ca. 500 nm. These cells are probably the structural elements of the fractal at the 2nd level of the hierarchy. Thus, the size of such a fractal is {(1.95 + 2)/1.95}^0.5^·1.59 = 2.26 μm. It represents a “drag-net”, in other words, a “seine” net, which consists of the above-mentioned cells with a characteristic size of 500 nm.

As can be seen from the SEM images (Figure 3a and Figure S2a in Supplementary Materials), the morphology of the air-dried NGF of BC constitutes a dense network [50] with BC nanoribbons tightly adhering to each other. The nanoribbons themselves are in the order of micrometers long, representing nanofibrils assembled in bundles. In width, the nanoribbons vary up to hundreds of nanometers. It can be seen in the micrograph that the nanoribbons are twisted along their lengths. This allows one to estimate their thickness to be a few tens of nanometers. Figure 4a shows several structural elements obtained from VSANS/SANS for the NGF of bacterial cellulose in a native state. Therefore, the SAS data obtained are in good agreement with the electron microscopic study.

Based on the microphotographic and USANS/SANS data presented, one can think that in the native state, the nano-gel film has a “drag-net” structure in contrast to the air-dried state. When the network is formed, channels appear in the BC, which could be 20 to 50 μm wide. The bacteria can move freely through these channels. On the other hand, this “drag-net”, which is a nano-gel film, is filled with water to the extent that the dry residue/water ratio is 1/100. In other words, since there are two orders of magnitude more water in native cellulose than in dry matter, the nano-gel film is essentially a “trapped” water or hydrogel.

### 4.2. Interpenetrating Polymer Networks Based on BC and PVT

It should be noted that for the air-dried composite hydrogel of PVT/BC in comparison with dry BC (Figure 3a,b), cellulose nanoribbons cannot be directly distinguished in the micrographs. The point is that in the dry residue, there is only 4–5 weight % cellulose in relation to the remaining PVT (Table 1). Moreover, X-ray diffractograms of air-dried BC/PVT composites did not reveal the characteristic diffraction reflections of cellulose I_α_ with a one-chain triclinic P1 unit cell [44], observing only a single polymeric amorphous halo (Figure S4b,c in Supplementary Materials). The polymeric network consisting of MBA nodes and linear fragments of VT structural units initially occupies lacunae in the BC hydrogel, preventing them from collapsing during air drying, as happens in the case of BC nano-gel film. The presence of the initial lacunae in the IPN is evident in the SEM micrographs at lower magnifications (Figure 3c,d). The lacunae boundaries appear as “wirings” on the surface of the composite particles (air-dried hydrogel).

**Figure 5:**
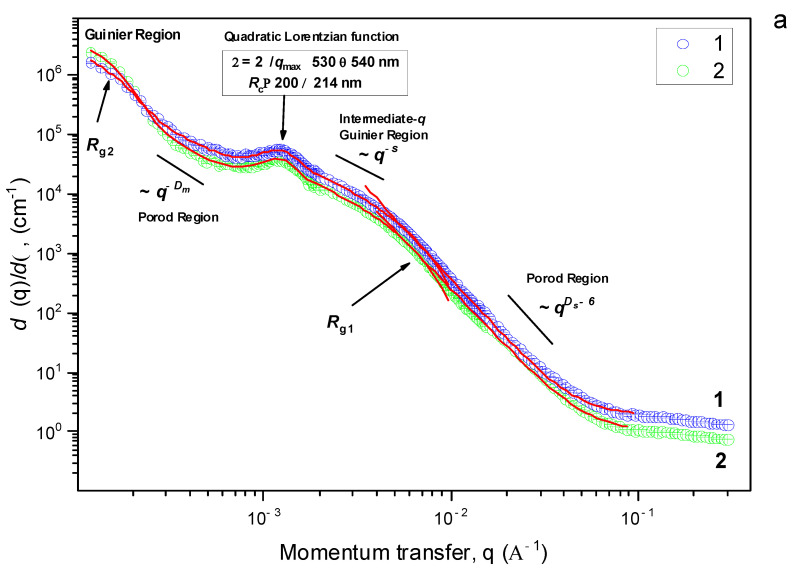

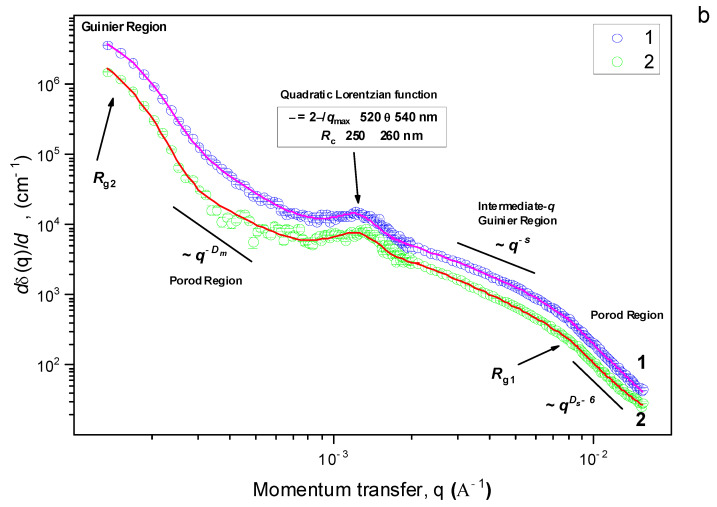
SANS/VSANS data for interpenetrating polymer networks representing composite hydrogels based on BC and PVT (**a**) and PVT/oPA complex (**b**). Blue color (1) depicts the MBA concentration of 0.2 mol.%, and green (2) represents the MBA concentration of 0.5 mol.%. The red lines are the result of fitting the SAS data.

To investigate the undistorted supramolecular structure of the composite hydrogel, we also applied the method of small-angle neutron scattering with the use of contrast variation by replacing H_2_O with D_2_O. The *q*-dependences of the SANS/VSANS cross-sections for the interpenetrating polymer networks based on BC and PVT are presented in Figure 2a,b in the log-log scale. The SANS/VSANS results for BC–PVT hydrogels without H_3_PO_4_ and with the PVT/ H_3_PO_4_ complex are summarized in Table 2. The analysis of SANS/VSANS curves showed, as before (Figure 2), the presence of two structural levels and a correlation peak (Figure 5).

The first structural level is the lamellae (Figure 4b). In cross-section, a lamella has a rectangle with a thickness characterized by a radius of gyration, *R*_g1_, from 12 to 21 nm and a width, *R*_c_, from 138 to 260 nm (Table 2). How is this width found? The point is that we found on the SANS/VSANS curves for our interpenetrating networks of BC–PVT and native BC hydrogels the presence of a distinct correlation peak at *q*_max_ = ~0.00125 Å^−1^. This peak was treated using the quadratic Lorentzian function (see Equation (S1) in Supplementary Materials). This indicates the presence of near structural order in hydrogels with characteristic distance of ξ *=* 2*π/q_max_* ≃ (520 ÷ 540) nm (Table 2).

Moreover, the width of this peak, *R*_c_, gives, in our opinion, the width of the overlapping lamellae that forms the near order. Thus, this near order is related to the structure of native bacterial cellulose.

The authors of [24] observed a drop in the same region of momentum transfer where we see a correlation peak. This may well be because the slit geometry with a double crystal technique was used in [24] as opposed to the pinhole geometry on KWS-3. The drop is in the region of large momentum transfer for the double crystal technique, where the signal-to-noise ratio is small for peak resolution.

It can be seen that in small *q* at the second structural level (Table 2), linear dependencies on a double logarithmic scale are observed (Table 2), with a slope of *m*_2_ for the curves from 1.95 to 2.86. A model of the Porod exponent, *m*_2_, in the interval of minimum transmitted pulses was used. The scattering intensity *I*(*q*) is calculated as the quadratic Lorentzian [49] (see Equation (S4) in Supplementary Materials).

In this interval of small momentum transfer, *q*, one can talk about a volume fractal structure, which can be a polymer network consisting of cellulose lamellae (nanoribbons), the space between which is filled with cross-linked polymeric coils of PVT, occupying the space in huge lacunae with a diameter larger than the near order in (500 ÷ 540) nm (Figure 4b). It was found that the IPN becomes denser in comparison with native BC. For example, it can be seen that the slope *n*_2_ increases from 1.95 (native BC) to 2.52–2.86 in the system with PVT.

In the region of momentum transfer, *q*, larger than ~0.002 Å^−1^ we used the generalized Guinier-Porod model [51]. A value of *s* close to 2 for native BC indicates that lamellae (nanoribons) scatter. However, the value of s decreases to 1.34 when passing to the IPN, indicating that the scattering objects are rod shaped. It can be assumed that PVT envelops BC nanoribbons, forming an interpolymer complex with them due to hydrogen bonds. The value of the Porod exponent, *m*_1_, for the 1st structural level of native BC is less than 3 (Table 2), which indicates a volume fractal distribution of the mass centers of crystallites of nanoribbons and their fluctuation: fractal dimension *D_v_* = *m*_1_. This behavior is consistent with the data in [24]. However, in the case of IPC, the value of *m*_1_ becomes larger than 3. This means that the nanoribbons are compacted by interaction with the internal network formed by the PVT. This may be in the case of complex formation due to hydrogen bonding between BC and PVT as a result of envelopment of nanoribbons by polymer chains of PVT, as mentioned above. One can talk about the formation of surface fractal structures, and the fractal dimension is *D_s_* = 6 − *m*_1_ (Table 2).

## 5. Conclusions

The data obtained from SANS and VSANS allow us to state that the native (pristine) BC has a two-level hierarchical organization of SMS in the mesoscopic size range. At the first level, neutron scattering was observed on the section of lamellae, which are BC nanoribbons consisting of microfibrils’ bunches. In addition, a significant result was obtained: pristine BC has the peak in the SANS/VSANS curve characterizing near-range order to ca. 500 nm. At the second hierarchical level of the NMS organization in the range of more than 2 μm one can talk about a volume fractal with dimensionality 1.95, which indicates the proximity to the Gaussian distribution of dry crystalline matter packing in the nano-gel film. This is in good agreement with the high water content of the nano-gel film and its good sorption properties. Furthermore, the hierarchical organization for BC’s supramolecular structure presented in this paper coincides with Brown’s BC model, which was earlier supported by Fink’s results [11,12].

In IPN, an interpolymer complex could be formed between the BC lamellae and PVT chain segments. As a result, compactization of the polymeric substance inside the IPN takes place. One can think that, at different types of BC treatment, when one or another type of drying (dehydration) is used, it is possible to observe the disappearance of the near-order-collapse peak of the BC nano-gel film, as it happens during air drying. At the same time, the behavior of the slope of the SAS curve, which characterizes the fractal dimensionality in the region of momentum transfer in the ultra-small-angle range, is also interesting for the study of BC-based composites, such as IPN. In other words, it is necessary to verify how stable the elements of bacterial cellulose SMS are to different types of treatment, and the peak found on the SAS curve can be a good reference to verify the nativity of the nano-gel film, and thus its invariance can be a criterion to test possible hypotheses. The results obtained are important in the context of nanotechnology for regenerative medicine, lightweight materials, optoelectronics and membrane technology.

## Figures and Tables

**Table 1:** Composition and characteristics of composite hydrogels. The swelling time is 8 h in the reaction mixture. The AIBN concentration is 0.5 wt%.

Sample	MBA, mol.%	H_2_O Content in Hydrogel, wt%	BC Content, ^(1)^ wt%	PVT Content, ^(1)^ wt%	Degree of Equilibrium Swelling, g/g	H_3_PO_4_ Content, ^(1)^ wt%
**1**	0.2	48.0	4.0	96.0	0.9	-
**2**		61.3	0.9	22.4	1.6	76.7
**3**		53.1	2.9	72.3	1.1	24.8
**4**	0.5	33	5.1	94.9	0.5	-
**5**		19.7	1.8	33.3	0.24	64.9
**6**		13.1	4.1	72.8	0.15	23.1

Note: ^(1)^ Weight percent in the dry residue of the air-dried to constant weight hydrogel.

**Table 2:** Micro- and mesostructure parameters of native BC and hydrogels of BC/PVT and BC/PVT//H_3_PO_4_, obtained from the analysis of SANS/VSANS data.

Samples	BC	BC/PVT		BC/PVT/H_3_PO_4_	
% MBA	n/a	0.2	0.5	0.2	0.5
2 structural level ^(1)^					
*R*_g2_ (μm)	1.59 ± 0.05	1.36 ± 0.05	1.42 ± 0.08	1.41 ± 0.06	1.51 ± 0.07
*m*_2_	1.95 ± 0.08	2.58 ± 0.08	2.85 ± 0.06	2.86 ± 0.09	2.52 ± 0.06
1 structural level ^(2)^					
*R*_c_ (nm) ^(3)^	138 ± 4	199 ± 10	214 ± 10	259 ± 17	256 ± 17
ξ = 2π/*q*_max_(nm)	507 ± 4	537 ± 5	528 ± 4	523 ± 6	537 ± 5
*s* ^(4)^	2.06 ± 0.02	1.37 ± 0.02	1.56 ± 0.02	1.34 ± 0.02	1.42 ± 0.02
*R*_g1_ (nm)	12.1 ± 0.5	19.2 ± 0.5	20.4 ± 0.5	15.7 ± 0.5	15.0 ± 0.7
*m*_1_	2.56 ± 0.03	3.41 ± 0.03	3.32 ± 0.03	3.66 ± 0.06	3.47 ± 0.02
*D_m_* ^(5)^	2.56	–	–	–	–
*D_s_* ^(6)^	–	2.59	2.68	2.34	2.53
*T = R*_g1_√12 (nm) ^(7)^	42	67	71	54	52

Note: ^(1)^ Standard Guinier/Porod law formula is used when s = 0 (see Equation (S1) in Supplementary Materials). ^(2)^ Generalized Guinier/Porod approximation is used (see Equation (S1) in Supplementary Materials). ^(3)^ Quadratic Lorentzian function is used (see Equation (S4) in Supplementary Materials). ^(4)^ *s* is the dimension variable (see Equation (S1) in Supplementary Materials). ^(5)^ *D_m_* is fractal dimension for mass (volume) fractal; ^(6)^ *D_s_* is fractal dimension for the surface fractal, *D_s_* = 6 − *m*_1_; ^(7)^ T is lamella (nanoribbon) thickness.

## Data Availability

Data are contained within the article and Supplementary Materials.

## Supplementary Materials

The following supporting information can be downloaded at: https://www.mdpi.com/article/10.3390/biomimetics8070520/s1, Figure S1. The SEM microphotograph for an air-dried nano-gel film of BC (a) and a composite of BC and PVT (b), Figure S2. The SEM microphotograph for a composite of BC and PVT (a) and element distribution plot for this composite (b). Orange stands for C, green does for N, and lilac for O, Figure S3. The mapping SEM microphotograph for a composite of BC and PVT with color highlighting (a) and the element distribution maps for the selected region (b). Orange (C-KA)) stands for C, green (N-KA) does for N, and lilac (O-KA) for O, Figure S4. XRD an air-dried nano-gel film of BC (a), PVT (b), and a composite of BC and PVT (c), Table S1. Element distribution for a composite of BC and PVT.
