# Supplementary material for: Composite Hydrogels Based on Bacterial Cellulose and Poly-1-vinyl-1,2,4-triazole/Phosphoric Acid: Supramolecular Structure as Studied by Small Angle Scattering
Source: Biomimetics (Basel). 2023 Nov 2;8(7):520. doi: 10.3390/biomimetics8070520 (PMC10669583; doi:10.3390/biomimetics8070520)
Supplement: Supplementary file 1 [file biomimetics-08-00520-s001.zip › biomimetics-2615200-supplementary.pdf]

biomimetics

MDPI

Article

Electronic Support Materials:

Composite Hydrogels Based on Bacterial Cellulose and Poly-1-vinyl-1,2,4-triazole/Phosphoric Acid: Supramolecular Structure as Studied by Small Angle Scattering

Ruslan Y. Smyslov [1,2], Artem I. Emel'yanov [3], Ksenia V. Ezdakova [2], Svetlana A. Korzhova [3], Yulia E. Gorshkova [4,5], Albert K. Khripunov [1], Alexandra V. Migunova [6], Natalia V. Tsvigun [7], Galina F. Prozorova [3], Varvara O. Veselova [8], Gennady P. Kopitsa [2,9], Lijun Lu [10], Yanchao Mao [10] and Alexander S. Pozdnyakov [3,*]

[1] Institute of Macromolecular Compounds RAS, NRC KI, 199004 Saint Petersburg, Russia; urs@macro.ru (R.Y.S.); biocell@macro.ru (A.K.K.)
[2] Petersburg Nuclear Physics Institute NRC KI, 188300 Gatchina, Russia; matissa.vkv@gmail.com (K.V.E.); kopitsa_gp@pnpi.nrcki.ru (G.P.K.)
[3] A.E. Favorsky Irkutsk Institute of Chemistry, Siberian Branch, Russian Academy of Sciences, 664033 Irkutsk, Russia; emelyanov@irioch.irk.ru (A.I.E.); korzhova@irioch.irk.ru (S.A.K.); prozorova@irioch.irk.ru (G.F.P.)
[4] Joint Institute for Nuclear Research, 141980 Dubna, Russia; yulia.gorshkova@jinr.ru
[5] Institute of Physics, Kazan Federal University, 420008 Kazan, Russia
[6] Department of Microbiology, Biological Faculty, Saint Petersburg State University, 199178 Saint-Petersburg, Russia; sasha_mig_2405@mail.ru
[7] Federal Scientific Research Centre "Crystallography and Photonics" of the Russian Academy of Sciences, 111933 Moscow, Russia; n_tsvigun@mail.ru
[8] Kurnakov Institute of general and inorganic chemistry RAS, 119991 Moscow, Russia; ibvarvara@yandex.ru
[9] I.V. Grebenshchikov Institute of Silicate Chemistry RAS, NRC KI, 199034 Saint Petersburg, Russia
[10] Key Laboratory of Materials Physics of Ministry of Education, School of Physics and Microelectronics, Zhengzhou University, Zhengzhou 450001, China; ymao@zzu.edu.cn (Y.M.)
* Correspondence: pozdnyakov@irioch.irk.ru

1. SANS and VSANS studies of native BC

The q-dependences of the SANS/VSANS cross-sections for the NGF of native BC are presented in Figure 2 (in the paper body text) in log-log scale. As can it be seen from this figure, the neutron scattering curves of the native cellulose show two sections obeying the power law and a well-defined peak.

In SANS experiment, the power-law dependence of cross section is observed in a certain q-region q>1/R, where R is the characteristic scale of a structure level of the system. At this time, its fractal dimension is determined by the exponent of power m, or, more precisely, by the deviation from the Porod asymptotic dependence (m = 4) [S1]. Meanwhile, the crossover point q_c allows to calculate the characteristic scale R of scattering heterogeneity of observed structure level. It could be done by using the Guinier approximation [S2,S3].

Generalized Guinier/Porod approximation

For an approximation of the SANS data, we used an empirical model [S4], in which multiple Guinier and Porod regions can be identified, as:

$$I(Q) = \begin{cases} G\,Q^{-s} exp\left(-\frac{R_g^2 Q^2}{3-s}\right) & Q \leq Q_1 \\ DQ^{-m} & Q \geq Q_1 \end{cases}, \qquad \text{(S1)}$$

where G is the exponential (Guinier) prefactor, s is a dimension variable, R_g is a Radius of gyration, m is a Porod exponent. I

The prefactor D, and together with the quantity Q_1, are obtained from the continuity conditions of the Guinier and Porod terms as well as of their derivatives. They can be written explicitly as:

$$Q_1 = \frac{1}{R_g}\sqrt{(m-s)(3-s)/2}$$

and respectively:

$$D = G\,exp\left(-\frac{R_g^2Q^2}{3-s}\right)Q_1^{m-s}.$$

The first term in Eq. E1 is the **generalized Guinier law**, which is a determining method for calculating the sizes of scattering objects of any shape in the region of small Q ($QR_g < 1$). For 3D globular objects (such as spheres), s = 0, and one recovers the standard Guinier formula. For 2D symmetry (such as for rods) s = 1, and for 1D symmetry (such as for lamellae or platelets) s = 2 [S5,S6]. The fractal dimension, D_m, and equilibrium structure qualities were evaluated from a relatively larger Q region using the Porod low – the second term in Eq. S1

Unified global scattering function

The data from VSANS, SANS and SAXS demonstrate a behavior that is typical for scattering of systems with hierarchical multilevel structure [S3,S7]. In such structure, large particles are formed by smaller particles or systems consisting of a few different types of heterogeneities. Therefore, for the analysis of neutron data the following formula was used in the frame of the unified global scattering function for three structural levels, and for the general case can be written as [S3,S8]:

$$\frac{d\Sigma(q)}{d\Omega} = \sum_{i=1}^{m}(G_i\cdot exp\left(-\frac{q^2R_{gi}^2}{3}\right) + B_i\,exp\left(-\frac{q^2R_{g(i-1)}^2}{3}\right)\left[\frac{(erf(qR_{gi}/\sqrt{6}))^3}{q}\right]^{n_i}) + I_{inc}, \quad \text{(S2)}$$

where i is the hierarchical level in the system (from 0 to m) [S8]. In the common case formula (6) stipulates availability of four free arguments for each structure level: R_{gi} – radius of gyration of ith level, n_i – power exponent which corresponds to the fractal dimension of i-level heterogeneity, G_i – Guinier prefactor, B_i – power prefactor. The experimental curves of the differential cross sections $d\Sigma(q)/d\Omega$ versus q were fitted by the least mean squares method over the entire measured q range. Variables q and q^* are renormalized using the error function, $erf(x)$, in the power laws as follows:

$$q_i^* = q/[\text{erf}\,(kqR_{g,i}/\sqrt{6})]^3. \quad \text{(S3)}$$

This procedure allows one to correctly describe the behavior of the neutron scattering intensity, $I(q)$, in the "intermediate" interval between $qR_q < 1$ (Guinier approximation) and $qR_g >> 1$ (asymptotic q^{-n}). The experimental data are approximated using the method of least squares.

Quadratic Lorentzian function

It can be seen that in small q at the second structural level (see Tables 1 and 2 in the main paper), linear dependencies on a double logarithmic scale are observed (see Tables 1 and 2), with the slope of n_2 for the curves from 1.95 to 2.86. The model of the Porod exponent, n_2, in the interval of minimum transmitted pulses was used. The scattering intensity $I(q)$ is calculated as [47]

$$I(q) = A/(qR)^m + C/[1 + (|q - q_{max}|R_c)^2]^2 + B, \quad \text{(S4)}$$

where A is the Porod law scale factor; m is the Porod exponent; R is correlation size of Porod-law scattering inhomogeneities; C is the Lorentzian scale factor; R_c is the screening length, and B is the flat background.

2. SEM

As can be seen from the SEM images in Figure S1a, the morphology of the air-dried NGF of BC constitutes a dense network [46] with BC nanoribbons tightly adhering to each

other. The nanoribbons themselves are on the order of micrometers long, representing nanofibrils assembled in bundles. In width, the nanoribbons vary up to hundreds of nanometers. It can be seen in the micrograph that the nanoribbons are twisted along their length. This allows ones to estimate their thickness to be a few tens of nanometers. Figure 3a (in the main paper) shows the several structure elements obtained from VSANS/SANS for the NGF of bacterial cellulose in a native state. So, the SAS data obtained are in good agreement with the electron microscopic study.

Figure S1b shows a surface micrograph of the BC/PVT composite obtained by drying the corresponding hydrogel.

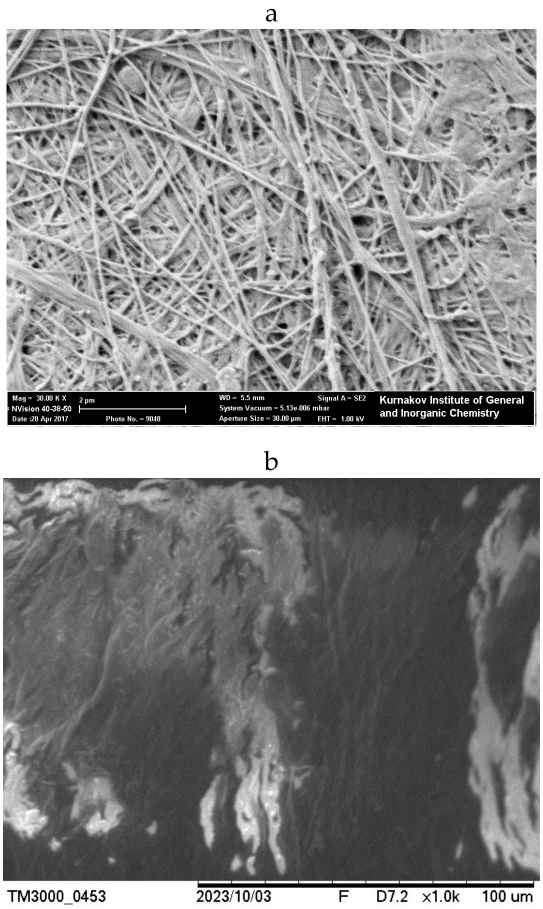

Figure S1. The SEM microphotograph for an air-dried nano-gel film of BC (a) and a composite of BC and PVT (b).

Based on SEM image mapping data by EDX method (Figure S2), the elemental composition of BC/PVT composites obtained by air-drying from hydrogels was calculated. Table S1 shows the results of the elemental composition calculation for the elements: C, N and O from the distribution (Fig. S2b).

a

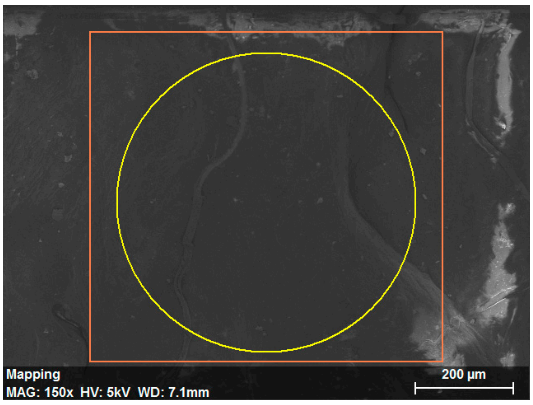

b

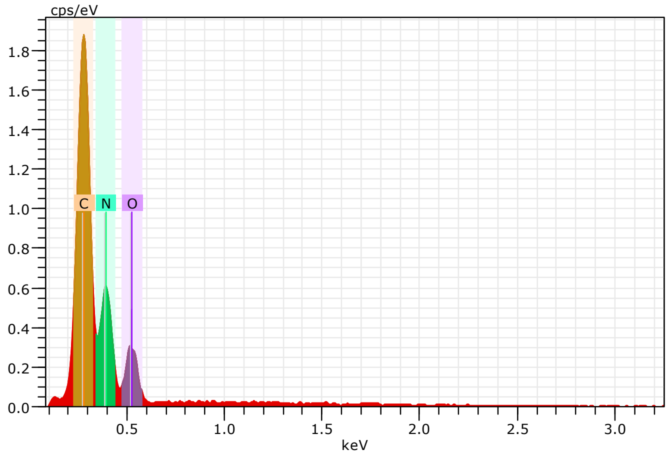

Figure S2. The SEM microphotograph for a composite of BC and PVT (a) and element distribution plot for this composite (b). Orange stands for C, green does for N, and lilac for O.

Table S1. Element distribution for a composite of BC and PVT

Element	Series	Norm. wt.%	Atom. wt.%
Carbon	K	45.1	49.7
Nitrogen	K	40.4	38.2
Oxygen	K	14.6	12.1

Figure S3 shows the mapping results for each of the three elements: C, N and O for the BC/PWT composite for the selected site (Fig. S2a).

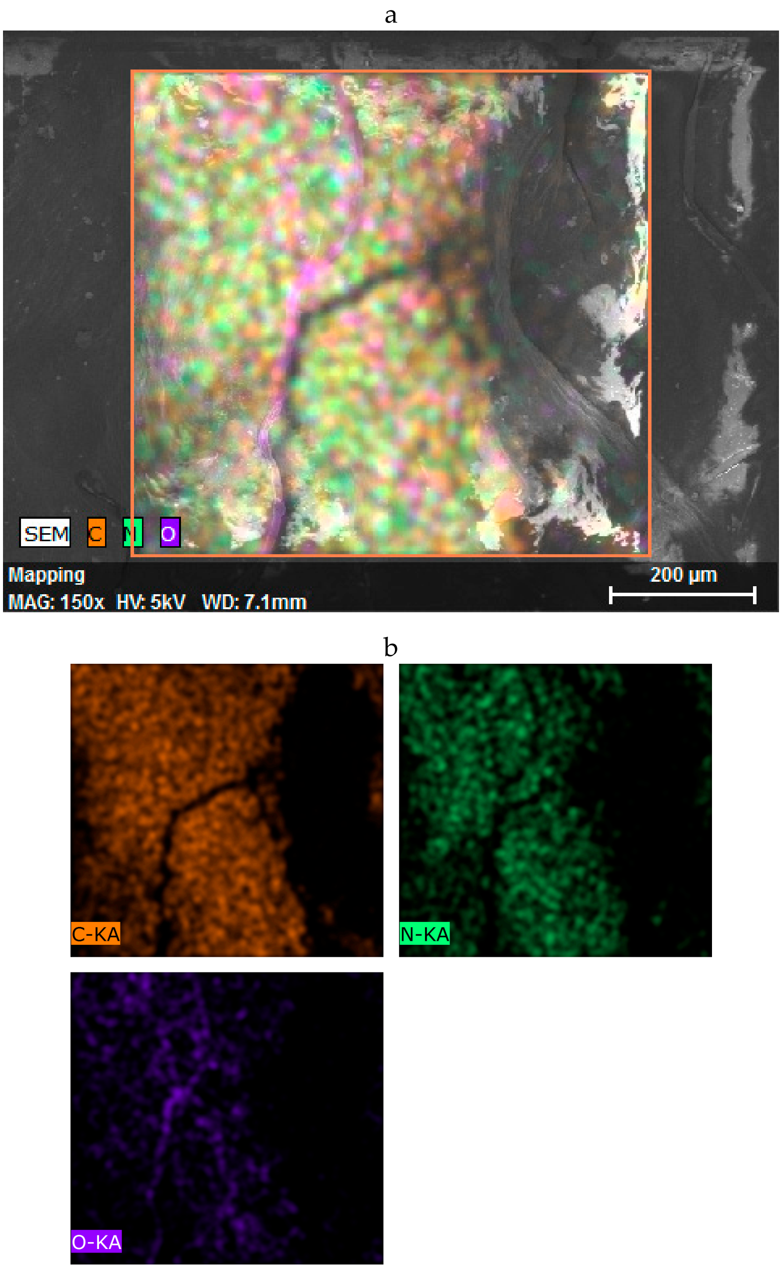

Figure S3. The mapping SEM microphotograph for a composite of BC and PVT with color highlighting (a) and the element distribution maps for the selected region (b). Orange (C-KA)) stands for C, green (N-KA) does for N, and lilac (O-KA) for O.

2. XRD for composite nanogels

Air-dried native bacterial cellulose nano-gel film was investigated by XRD method (Fig. S4). BC displays characteristics of highly crystalline, Iα-rich cellulose [S9]. Iα corresponds to a one-chain triclinic P1 unit cell: a = 6.717Å, b = 5.962 Å, c = 10.400 Å, α = 118.08°, β = 114.80°, and γ = 80.37° (4114382.cif store at COD) [S10].

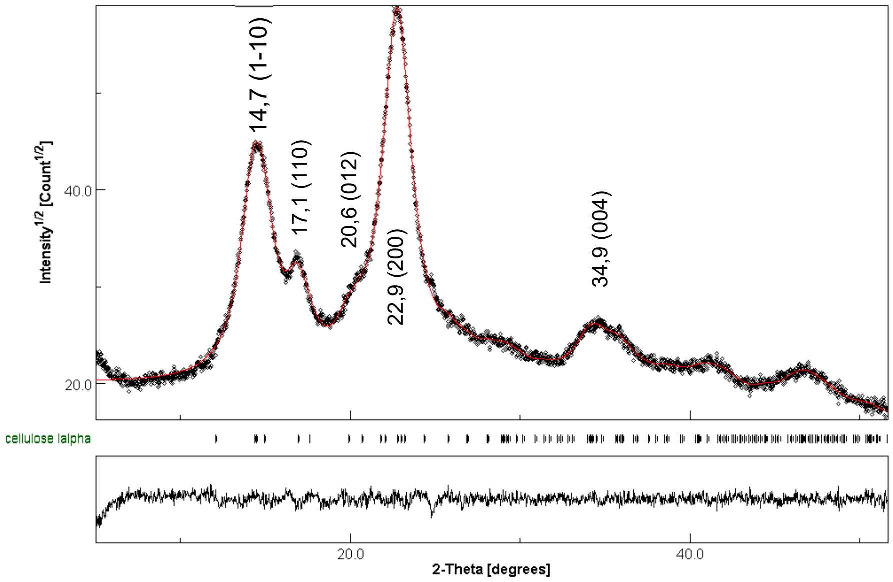

(a)

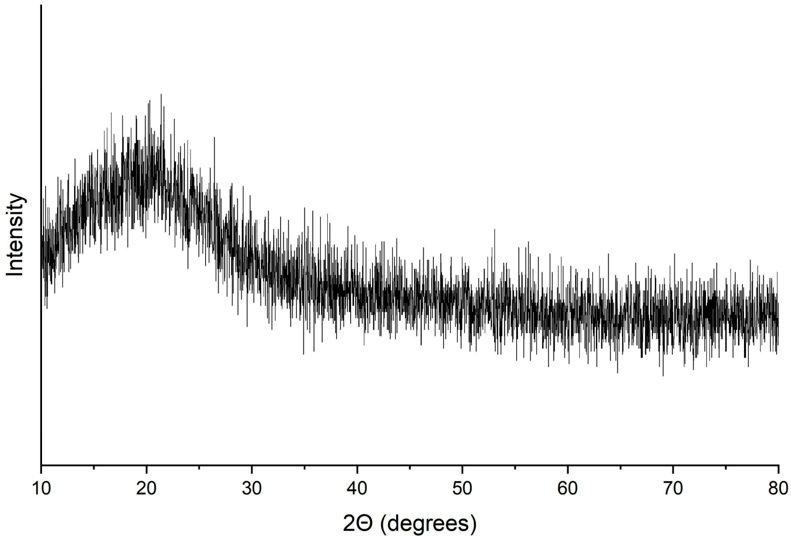

(b)

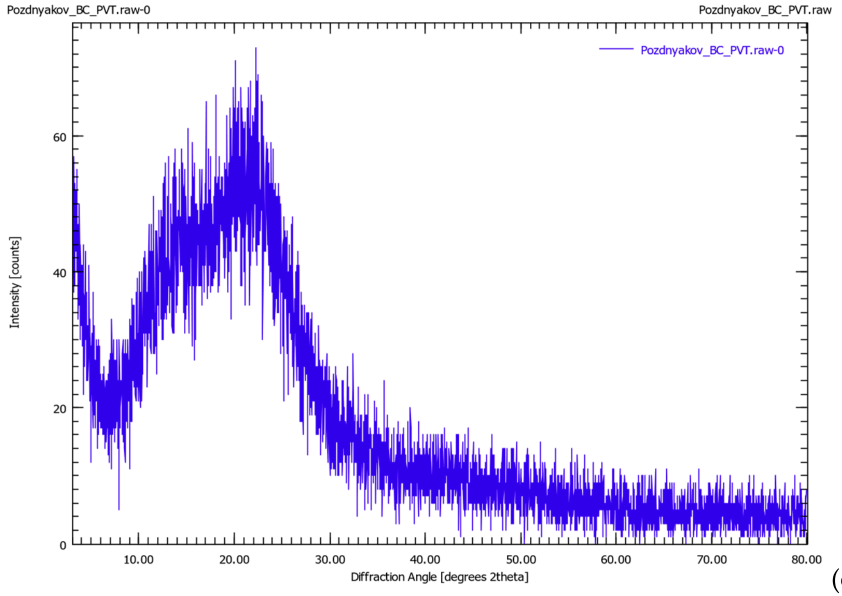

(c)

Figure S4. XRD an air-dried nano-gel film of BC (a), PVT (b), and a composite of BC and PVT (c).

X-ray diffraction for the PVT/BC composite obtained by air drying of the composite hydrogel is shown in Fig. S4c. Against the background of the amorphous signal from the PVT film (Fig. S4b), the presence of crystalline BC reflections (Fig. S4a) is hardly visible due to the insignificant by volume cellulose content in the composite – about 4%.

References

S1. Teixeira, J. Experimental Methods for Studying Fractal Aggregates. In *On Growth and Form*; Springer Netherlands: Dordrecht, 1986; pp. 145–162.

S2. Guinier, A. La diffraction des rayons X aux très petits angles: application à l'étude de phénomènes ultramicroscopiques. *Ann. Phys. (Paris).* **1939**, *11*, 161–237, doi:10.1051/anphys/193911120161.

S3. Beaucage, G.; Schaefer, D.W. Structural studies of complex systems using small-angle scattering: a unified Guinier/power-law approach. *J. Non. Cryst. Solids* **1994**, *172–174*, 797–805, doi:10.1016/0022-3093(94)90581-9.

S4. Hammouda, B. A new Guinier–Porod model. *J. Appl. Crystallogr.* **2010**, *43*, 716–719, doi:10.1107/S0021889810015773.

S5. Hjelm Jnr R.P., Thiyagarajan P., Sivia D.S., Lindner P., Alkan H. and Schwahn D., Small-angle neutron scattering from aqueous mixed colloids of lecithin and bile salt, *Prog. Colloid Polym. Sci.*, 1990, 81: 225–231. DOI: 10.1007/bfb0115557.

S6. Hjelm Jnr R.P., Thiyagarajan P. and Alkan H., A small-angle neutron scattering study of the effects of dilution on particle morphology in mixtures of glycocholate and lecithin, *J. Appl. Cryst.*, 1981, 21: 858–863. DOI: 10.1107/S002188988800531X.

S7. Štěpánek, M.; Matějíček, P.; Procházka, K.; Filippov, S.K.; Angelov, B.; Šlouf, M.; Mountrichas, G.; Pispas, S. Polyelectrolyte−Surfactant Complexes Formed by Poly[3,5-bis(trimethylammoniummethyl)4-hydroxystyrene iodide]- block -poly(ethylene oxide) and Sodium Dodecyl Sulfate in Aqueous Solutions. *Langmuir* **2011**, *27*, 5275–5281, doi:10.1021/la200442s.

S8. Beaucage G (2012) 2.14 - Combined Small-Angle Scattering for Characterization of Hierarchically Structured Polymer Systems over Nano-to-Micron Meter: Part II Theory. In: Matyjaszewski K, Möller M (eds) Polymer Science: A Comprehensive Reference. Elsevier, Amsterdam, pp 399–409.

S9. VanderHart, D. L., & Atalla, R. H. (1984). Studies of microstructure in native celluloses using solid-state carbon-13 NMR. *Macromolecules*, 17(8), 1465–1472. doi:10.1021/ma00138a009

S10. Stephanie Hesse-Ertelt, Thomas Heinze, Eiji Togawa, Tetsuo Kondo.Structure elucidation of uniformly 13C-labeled bacterial celluloses from different *Gluconacetobacter xylinus* strains. Cellulose (2010) 17:139–151. DOI 10.1007/s10570-009-9355-4.
